# Use of electronic cigarettes and heated tobacco products during the Covid-19 pandemic

**DOI:** 10.1038/s41598-021-04438-7

**Published:** 2022-01-13

**Authors:** Silvano Gallus, Chiara Stival, Giulia Carreras, Giuseppe Gorini, Andrea Amerio, Martin McKee, Anna Odone, Piet A. van den Brandt, Lorenzo Spizzichino, Roberta Pacifici, Alessandra Lugo

**Affiliations:** 1 Department of Environmental Health Sciences, Istituto Di Ricerche Farmacologiche Mario Negri IRCCS, Via Mario Negri 2, 20156 Milan, Italy; 2 Oncologic Network, Prevention and Research Institute (ISPRO), Florence, Italy; 3 Department of Neuroscience, Rehabilitation, Ophthalmology, Genetics, Maternal and Child Health, Section of Psychiatry, University of Genoa, Genoa, Italy; 4 IRCCS Ospedale Policlinico San Martino, Genoa, Italy; 5 Centre for Global Chronic Conditions, London School of Hygiene and Tropical Medicine, London, UK; 6 School of Medicine, University Vita-Salute San Raffaele, Milan, Italy; 7 Department of Public Health, Experimental and Forensic Medicine, University of Pavia, Pavia, Italy; 8 Department of Epidemiology, GROW- School for Oncology and Developmental Biology, Maastricht University Medical Centre, Maastricht, The Netherlands; 9 Department of Epidemiology, CAPHRI- School for Public Health and Primary Care, Maastricht University Medical Centre, Maastricht, The Netherlands; 10 Ministry of Health, Rome, Italy; 11 National Centre On Addiction and Doping, Istituto Superiore Di Sanità, Rome, Italy

**Keywords:** Public health, Epidemiology

## Abstract

Only a few studies investigated changes in electronic cigarette (e-cigarette) and heated tobacco product (HTP) use during pandemic restrictions. We conducted a web-based cross-sectional study of a representative sample of 6,003 Italian adults during the strictest phase of the Covid-19 lockdown (April–May 2020). Participants were asked to report changes in e-cigarette and HTP use compared to before the pandemic. E-cigarette users increased from 8.1% to 9.1% and HTP users from 4.0% to 4.5%. Among e-cigarette non-users before lockdown, 1.8% started using e-cigarettes during lockdown. New users were more frequently younger (p for trend 0.001), men (odds ratio, OR 1.56; 95% confidence interval, CI: 1.03–2.34), cannabis users (OR 2.35; 95% CI: 1.33–4.13), gamblers (OR 3.34; 95% CI: 2.18–5.11) and individuals with anxiety symptoms (OR 1.58; 95% CI: 1.00–2.52). 1.0% of HTP non-users started using it during lockdown. New users were less frequently current than never cigarette smokers (OR 0.19; 95% CI: 0.06–0.61) and more frequently gamblers (OR 2.23; 95% CI: 1.22–4.07). E-cigarettes and HTPs played little role as smoking cessation tools for hardcore smokers but rather provided opportunities for young never smokers to engage in socially acceptable activities, perhaps reflecting the obstacles they faced in obtaining other addictive substances during confinement.

## Introduction

Italy was the first European country to experience the full force of the pandemic. On 9 March 2020 its government imposed a nationwide “lockdown” to interrupt transmission of the coronavirus^1^: most workplaces and public places, including shops, bars and restaurants, closed and people were forbidden to leave their homes except to obtain basic necessities and healthcare^2^. In the three weeks prior to 4 May 2020, the regime intensified, suddenly changing the lives of millions of Italians^2^. Yet many shops selling tobacco and electronic cigarettes (e-cigarette) were exempt from these restrictions, following normal working hours even during the strictest phase of the lockdown. We now know that Covid-19 had a huge impact on several addictive behaviours among Italian adults. Smoking intensity increased substantially^3^, with commentators invoking the impact of confinement on mental health^4,5^.

Few studies have investigated the impact of the Covid-19 lockdown on the use of e-cigarettes and even fewer on the use of heated tobacco products (HTP). These studies—mainly small online surveys based on convenience samples—found a major impact on e-cigarette use, with 50–60% of vapers reporting changes in their use (either starting or quitting or increasing or decreasing consumption)^6–10^. To our knowledge, only two studies addressed the change in HTP use associated with the lockdown ^11,12^.

In Italy e-cigarette use has grown since 2010, and HTPs since 2016. The prevalence of regular e-cigarette use rose from 0.4% in 2014–2015 to 1.8% in 2016–2017 but fell back to 1.3% in 2018^13^. The market share of HTP among all legal tobacco products increased from 0.01% in 2015 to 0.67% in 2017 and to 4.33% in 2019^14^. An Italian web-based survey conducted in April 2020 (i.e., during the lockdown) on a convenience sample of 1825 subjects, found that both exclusive e-cigarette users and exclusive HTP users slightly increased their consumption during the lockdown^11^.

To our knowledge, no study has investigated the effect of the Covid-19 confinement on novel (tobacco) product use in adults using representative samples of the general population. For this purpose, the aim of this study is to evaluate the effects of Covid-19 lockdown on e-cigarette and HTP use in Italian adults, using data from a large representative cross-sectional study conducted within the LOckdown and lifeSTyles IN ITALY project^3,15^. Importantly, given evidence of the influence of industry links on research on this topic^16^, there is no conflict of interest in this study.

## Results

Of the sample of 6003 participants, 266 (4.4%) used e-cigarettes occasionally and 220 (3.7%) regularly before lockdown (early February 2020). The corresponding estimates during lockdown were 279 (4.7%) for occasional and 266 (4.4%) for regular use. Therefore, current e-cigarette users increased from 8.1% before lockdown to 9.1% during lockdown, making the relative increase 12.1%. Regular users increased by 20.9%. Similarly, 153 (2.6%) used HTPs occasionally and 87 (1.5%) used it regularly before lockdown and 147 (2.4%) used these products occasionally and 121 (2.0%) regularly during the lockdown. Current HTP users were 4.0% before and 4.5% during lockdown, with an 11.7% relative increase. Regular HTP users increased by 39.1%.

The apparent increase in HTP use in Italy is supported by official sales figures: compared to 2019, HTP sales in 2020 increased by 65%. In 2020, the market share of HTPs (7.5%) exceeded that of roll-your-own tobacco (7.0%), thus becoming the second most commonly sold tobacco product after conventional cigarettes (market share 81.5%).

Of 5516 e-cigarette non-users before lockdown, 98 (1.8%) started using e-cigarettes during lockdown (Table 1). These were more frequently men but initiation was less likely with increasing age (*p* for trend = 0.001). New e-cigarette users during lockdown were more frequently those who were cannabis users before the lockdown, gamblers, and those reporting anxiety symptoms. Of 5763 HTP non-users before lockdown, 55 (1.0%) started using them. The probability of initiation increased with level of education (*p* for trend 0.005), was less among those who smoked just before lockdown (compared to never smokers) and greater among gamblers.

**Table 1:** Distribution of non-users of e-cigarettes and heated tobacco products (HTP) according to starting their use during the Covid-19 lockdown, by selected demographic and socio-economic features, addictive behaviours and other individual characteristics. Corresponding odds ratios* (OR) and 95% confidence intervals (CI). Italy, 2020.

Characteristics (pre-lockdown)	E-cigarette non-users before lockdown	People who **start using e-cigarette** during lockdown		HTP non-users before lockdown	People who **start using HTP** during lockdown	
		%	OR (95% CI)		%	OR (95% CI)
Total	5516	1.8		5763	1.0	
**Sex**						
Women	2797	1.4	1.00^a^	2925	0.9	1.00^a^
Men	2720	2.2	**1.56 (1.03–2.34)**	2838	1.0	1.15 (0.67–1.97)
**Age group**						
18–34	1451	2.8	1.00^a^	1493	1.3	1.00^a^
35–54	2230	1.7	**0.64 (0.41–1.00)**	2342	1.0	0.77 (0.42–1.42)
55–74	1836	1.1	**0.39 (0.23–0.68)**	1928	0.6	0.49 (0.23–1.02)
P for trend			**0.001**			0.056
**Level of education**						
Low	850	1.4	1.00^a^	886	0.5	1.00^a^
Intermediate	2803	1.8	1.22 (0.65–2.30)	2922	0.7	1.25 (0.46–3.45)
High	1864	1.9	1.22 (0.63–2.37)	1954	1.5	**2.73 (1.03–7.30)**
P for trend			0.628			**0.005**
**Smoking status**						
Never	3758	2.0	1.00^a^	3887	1.2	1.00^a^
Former	477	2.1	1.25 (0.64–2.47)	537	0.8	0.74 (0.27–2.07)
Current	1281	1.1	0.59 (0.33–1.05)	1338	0.2	**0.19 (0.06–0.61)**
**Alcohol (AUDIT-C)**						
Not at risk	4087	1.6	1.00^a^	4257	0.8	1.00^a^
At risk	1430	2.3	1.49 (0.98–2.28)	1506	1.2	1.45 (0.82–2.55)
**Cannabis use**						
No	5162	1.6	1.00^a^	5376	0.9	1.00^a^
Yes	355	4.4	**2.35 (1.33–4.13)**	386	2.0	2.04 (0.93–4.50)
**Gambling**						
No	4647	1.3	1.00^a^	4847	0.8	1.00
Yes	870	4.6	**3.34 (2.18–5.11)**	916	1.8	**2.23 (1.22–4.07)**
**Anxiety symptoms (GAD-2)**						
No (score < 3)	4527	1.6	1.00^a^	4720	0.8	1.00^a^
Yes (score ≥ 3)	990	2.6	**1.58 (1.00–2.52)**	1043	1.5	1.78 (0.98–3.25)
**Depressive symptoms (PHQ-2)**						
No (score < 3)	4765	1.7	1.00^a^	4965	0.9	1.00^a^
Yes (score ≥ 3)	751	2.3	1.35 (0.79–2.28)	798	1.1	1.16 (0.55–2.43)

*Estimated by multiple logistic regression models after adjustment for sex, age, level of education and geographic area. Significant estimates at 0.05 level are in bold type.

^a^Reference category.

## Discussion

Our cross-sectional survey, based on a large representative sample of Italian adults, found that the prevalence of regular e-cigarette and HTP users rose following imposition of the first lockdown, by roughly 20% and 40%, respectively. A non-negligible number of Italian adults started using these novel (tobacco) products during the stay-at-home order. These were more frequently young adults, men, and those with certain addictive behaviours and mental health symptoms before lockdown.

Our findings are in line with another Italian survey among 1825 adults, which showed a slight increase in daily consumption among vapers during the lockdown^11^, although it should be borne in mind that the survey, which did not claim to be representative, was presented on the website of an organisation that has long promoted e-cigarettes, while one of the authors has close links to the tobacco industry^17^. Our findings are also consistent with evidence on adults from other countries—mainly from the USA and the UK. In general, the majority of adolescents or young adults reported less e-cigarette use during the stay-at-home order ^9,18–20^ whereas the majority of older adults reported moderate to large increases^8,10,21,22^.

Whereas the number of regular users largely increased, the prevalence of occasional users did not substantially change. This is likely due to the fewer opportunities during the pandemic to be involved in social events that might favour a sporadic use of these products. New e-cigarette users were not more frequently smokers of conventional cigarettes, and new HTP users were even more often never than current smokers. Once addicted to nicotine, these subjects often switch to conventional cigarette smoking, as shown in the few available longitudinal studies^23,24^.

Conversely, new users were more frequently people—mostly young men—with specific addictive behaviours that were inaccessible in Italy during the strictest phases of the lockdown, such as some types of gambling^25^, or that were unlikely to be acceptable to family members at home, such as cannabis use. This suggests that young adults, unable to satisfy certain addictions during the lockdown, might have attempted to compensate with these novel (tobacco) products. Our findings also point to the worrying link between cannabis and e-cigarette use reported in at least one study on US adolescents^26^.

New users were also more likely to report anxiety symptoms, and might have been encouraged to engage in e-cigarette use by stress and tension accentuated by the pandemic and lockdown^4^. In fact, an observational study showed that Twitter users who tweeted about e-cigarettes had more concerns than others about the pandemic^27^.

This first representative survey shows in only two to three months a substantial increase in the prevalence of use of e-cigarettes and HTPs likely due to the lockdown in Italy. According to the last report on global tobacco epidemic of the WHO, the observed trends are worrying from a public health perspective^28^. However, it is unclear whether the changes and trends observed in terms of novel (tobacco) product use will persist over time or they represent only the result of an extraordinary event. Therefore, future longitudinal studies are required to elucidate the issue. We found that in Italy, novel (tobacco) products rather than acting as smoking cessation tools for hardcore smokers, served more as socially acceptable opportunities for young never smokers and, in general, for people already dependent to other less socially acceptable addictive behaviors, who found themselves unable to engage in those habits during the stay-at-home order. Data show that addictive behaviors are closely related and therefore all the supporting programs for people with selected addictions should take into account the inter-relationship between such addictions and act in a joint way to avoid poly-dependence. In addition, in this critical period, psychological support is needed, not only for the mental well-being of people, but to avoid the onset of new addictive behaviors.

## Methods

We conducted a web-based cross-sectional study with a large representative sample of Italian adults aged 18–74 years (approximately 73% of the population). The interview was carried out during the strictest phase of the lockdown (between 27 April and 3 May 2020). Details on sampling methods are reported elsewhere^3,25^. Briefly, the survey was run by Doxa, the Italian branch of the Worldwide Independent Network/Gallup International Association. Subjects were randomly selected among the more than 140,000 Doxa online panel participants. A quota sampling method by age, sex and region (Nomenclature of Territorial Units for Statistics, NUTS, 2) was applied. In all, 6003 people (2962 men and 3041 women) participated.

The ethics committee (EC) of the Fondazione IRCCS Istituto Neurologico Carlo Besta approved the study protocol (File number 71–73, April 2020). All participants provided informed consent to participate. All methods were performed in accordance with the relevant guidelines and regulations of Scientific Reports and of Nature Research journals.

Recruited subjects completed an online self-administered questionnaire, giving information on demographic and socio-economic characteristics, such as level of education, selected addictive behaviours, including smoking conventional cigarettes^3^, cannabis use, alcohol use disorder according to the AUDIT-C scale^29^, and gambling^25^. Selected mental health indicators were obtained using validated scales, including anxiety levels (Generalized Anxiety Disorder scale, GAD-2)^30^, and depression (Patient Health Questionnaire, PHQ-2)^31^. Addictive behaviours and mental health indicators were reported before and during the lockdown.

All participants were asked to report their use of e-cigarettes and HTPs both before the Covid pandemic (reference time: early February 2020) and at the time of interview. E-cigarette use was assessed by asking subjects if they had ever tried, used in the past, occasionally used or regularly (i.e. daily) used electronic cigarettes. HTP use was determined by asking whether they knew about HTPs, if they had ever tried, used in the past, used occasionally or regularly (i.e. daily) HTPs, such as IQOS by Philip Morris or Glo by British American Tobacco.

We used official legal sales figures for HTPs and other tobacco products sold in Italy in 2020 to compute the contemporary market share of HTP tobacco. These data were obtained from the Italian Ministry of Finance^14,31^. Official sales data do not include electronic cigarettes since in Italy they are not classified as tobacco products.

### Statistical analysis

For each selected potential determinant, the odds ratios (OR) and corresponding 95% confidence intervals (CI) for subjects starting vs. those who did not start using e-cigarettes [or HTPs] during lockdown among e-cigarette [or HTP] non-users before lockdown, were estimated using multivariable logistic regression models after adjustment for sex, age group, level of education, and geographic area. The independent variables (i.e., potential determinants), including addictive behaviours and measures of mental health, referred to the period before lockdown (in early February 2020).

A statistical weight was applied to all the analyses to achieve representativeness of the national sample in terms of sex, age, socio-economic status, and geographic area. All analyses were done using SAS 9.4 (Cary, North Carolina, USA).

## Data Availability

Data that support the findings of this study and materials are available from the corresponding author, SG, upon reasonable request.
